# Erythritol, at insecticidal doses, has harmful effects on two common agricultural crop plants

**DOI:** 10.1371/journal.pone.0192749

**Published:** 2018-04-17

**Authors:** Sara E. Scanga, Bilal Hasanspahič, Edin Zvorničanin, Jasmina Samardžić Koženjić, Andrew K. Rahme, Jessica H. Shinn-Thomas

**Affiliations:** Department of Biology, Utica College, Utica, New York, United States of America; University of Vigo, SPAIN

## Abstract

Erythritol, a non-nutritive polyol, is the main component of the artificial sweetener Truvia^**®**^. Recent research has indicated that erythritol may have potential as an organic insecticide, given its harmful effects on several insects but apparent safety for mammals. However, for erythritol to have practical use as an insecticide in agricultural settings, it must have neutral to positive effects on crop plants and other non-target organisms. We examined the dose-dependent effects of erythritol (0, 5, 50, 500, 1000, and 2000 mM) on corn (*Zea mays*) and tomato (*Solanum lycopersicum*) seedling growth and seed germination. Erythritol caused significant reductions in both belowground (root) and aboveground (shoot) dry weight at and above the typical minimum insecticidal dose (500 mM erythritol) in tomato plants, but not in corn plants. Both corn and tomato seed germination was inhibited by erythritol but the tomato seeds appeared to be more sensitive, responding at concentrations as low as 50 mM erythritol (in contrast to a minimum damaging dose of 1000 mM erythritol for corn seeds). Our results suggest erythritol may have damaging non-target effects on certain plant crops when used daily at the typical doses needed to kill insect pests. Furthermore, if erythritol’s damaging effects extend to certain weed species, it also may have potential as an organic herbicide.

## Introduction

Erythritol (C_4_H_10_O_4_) is a polyol (sugar alcohol) that occurs naturally in some fruits and fermented foods [1,2]. Erythritol is one of more than a dozen different types of polyols that have been discovered in angiosperms. These sugar alcohols appear to function as osmoprotectants, antioxidants, and carbon-storage molecules within the plants [3–5]. Erythritol is also produced industrially for use as the main ingredient in the artificial sweetener Truvia^**®**^ [6], which also contains extract from stevia (*Stevia rebaudiana*) leaves (80–95% rebaudioside A; [7]) and unidentified “natural flavors” [6].

A recent article that reported toxic effects of erythritol on the common fruit fly *Drosophila melanogaster* [8] sparked interest in using this compound as an insecticide since it is recognized as safe for human consumption [1] but may kill certain insects [8–11]. Subsequent studies in the lab, greenhouse, and field revealed that erythritol (either alone or as a component of Truvia) has insecticidal effects on the oriental fruit fly (*Bactrocera dorsalis*) [12], spotted wing Drosophila (*D*. *suzukii*) [11,13–16], red imported fire ant (*Solenopsis invicta*) [17], and possibly on house flies (*Musca domestica*) ([18] but see [19]) (Table 1). The insecticidal effects were observed to be dose-dependent for *D*. *melanogaster* [8,10], *D*. *suzukii* [11,13], and *S*. *invicta* [17], but not for *B*. *dorsalis* [12] or *M*. *domestica* [18]. Generally, these results suggest that erythritol may have considerable promise as an insecticide, particularly in certified organic farming operations [11], and researchers continue to actively explore its effects on various insect pests.

**Table 1:** Comparison of minimum insecticidal dose of erythritol suggested by previous studies to minimum damaging dose to corn and tomatoes (present study).

**Organism**	**Source of erythritol**	**Erythritol concentrations examined (mM)**	**Minimum insecticidal dose of erythritol (mM)**	**Sources**
**Common fruit fly**, *Drosophila melanogaster*, Diptera: Drosophilidae	Erythritol	100–2000	500	[8]*
	Truvia	125–2000	500	[11]
	Erythritol	1000	1000	[9]
	Erythritol	500–2500	1000	[10]
**Spotted wing Drosophila**, *D*. *suzukii*, Diptera: Drosophilidae	Truvia	125–2000	500	[11]
	Erythritol	50–2000	500	[13]
	Erythritol	450–225188	450	[14]
	Truvia	500	500	[15]
	Erythritol	500–2000	500	[16]
**Oriental fruit fly**, *Bactrocera dorsalis*, Diptera: Tephritidae	Erythritol	8–2000	10	[12]
**House fly†**, *Musca domestica*, Diptera: Muscidae	Erythritol	500–2000	500	[18]
**Red imported fire ant**, *Solenopsis invicta*, Hymenoptera: Formicidae	Erythritol	8–1638	819	[17]
			**Minimum damaging dose (mM)**	
**Corn**				Present study
Seedlings	Erythritol	5–2000	---	
Seeds	Erythritol	5–2000	1000	
**Tomato**				Present study
Seedlings	Erythritol	5–2000	500	
Seeds	Erythritol	5–2000	50	

* also observed insecticidal effects of Truvia solution (concentration: 0.0952 g Truvia/ml; estimated erythritol concentration in Truvia solution: 771 mM)

† [19] also examined house fly survival after exposure to Truvia, but the erythritol was in solid form rather than in solution

In order for erythritol to have practical applications as an insecticide, it not only needs to kill target pests, but it should also have neutral to positive effects on non-target insects [16] as well as other organisms, including crop plants. Therefore, it is important to consider the effect of erythritol on crop plants, with the goal of determining the concentration of erythritol that can inhibit insect pests without negatively affecting crop productivity. To our knowledge, only one previous study has examined the effect of erythritol on plants. This study applied low levels of erythritol (ranging from 0.8 mM to 409 mM) to the growing media of garlic (*Allium sativum*) and the model plant *Arabidopsis thaliana*. Possible dose-dependent alterations to *Arabidopsis* seedling root growth and garlic bulb germination time and growth were found, with putatively inhibited growth at higher erythritol concentrations [20]. These results suggest that erythritol’s impact on crops may vary depending on concentration, but no studies have examined the effects of erythritol on common crop plants at the higher concentrations required to kill pest insects (e.g., ≥500 mM) [15] (Table 1). Furthermore, the impact to crop plants of spraying erythritol on aboveground stems and leaves is unknown.

The purpose of this study was to investigate the dose-dependent effects of erythritol on plant growth and seed germination in corn (*Zea mays*) and tomato (*Solanum lycopersicum*). These two plant species were chosen to evaluate potential differences between monocot (corn) and dicot (tomato) responses to erythritol. Both corn and tomatoes are among the top crop commodities produced worldwide according to the Food and Agriculture Organization of the United Nations [21]. We exposed corn and tomato seedlings and seeds to six concentrations (0, 5, 50, 500, 1000, 2000 mM) of erythritol, and examined the effects on growth and germination. Although corn plants did not respond to the erythritol treatments, we observed reduced tomato plant dry weight at and above 500 mM erythritol, which appears to be the typical minimum insecticidal dose [15] (Table 1). We also found negative dose-dependent effects of erythritol on seed germination for both crop species. These results suggest that erythritol may have potential as an herbicide, but when used as an insecticide, it may result in damaging non-target effects to plant crops.

## Methods

### Erythritol treatment conditions

Both seedlings and seeds were exposed to 6 different concentrations of erythritol (Honeyville^**®**^ Granular Erythritol, #77–126) in deionized water: 0 (control), 5, 50, 500, 1000, and 2000 mM. These erythritol concentrations were similar to the concentration ranges explored in previous studies, and the latter three concentrations were selected to correspond with concentrations found to be detrimental to insect pests [8] (Table 1). The single field trial to date used 500 mM erythritol spray prepared from Truvia Baking Blend [15].

### Seedling experiment

Corn (field corn, Carolina Biological Supply) and tomato (cv. Beefsteak, Plantation Products) plants were grown in a 1:1 mixture of Miracle Gro^**®**^ potting soil and sand in 4-inch circular pots (volume = 524 cm^3^). On 12 March 2017, seeds were assigned to pots at random and sown at 3 cm depth, then placed into a randomized complete block design under high intensity fluorescent bulbs (54W lights set to a 14/10 hr light/dark cycle) in a heated (~26 **°**C) greenhouse (n = 11 plants of each species per treatment group for a total of 66 corn plants and 66 tomato plants). The soil surface in each pot was watered daily at 09:00 hrs with tap water for the duration of the experiment. After emergence (8 days after planting), each seedling’s leaves were evenly sprayed daily at 15:00 hrs with 3 ml of the appropriate erythritol solution (the control plants were sprayed with 3 ml of deionized water). By day 21 of the experiment, the corn plants were harvested because they had grown so large that there was a risk they would start to compete for light. The tomato plants were allowed to grow to day 28 before harvesting.

After harvest, roots were first carefully cleaned of all soil material by rinsing them with tap water over 1.4 mm sieves. After rinsing, the roots were separated from the shoots by cutting at the base of the stem. Both roots and shoots were placed into a drying oven at 60°C for 48 hrs and then weighed on an analytical balance to the nearest 0.0001 g (S1 Dataset). Mean dry weight (biomass) of both roots and shoots was compared among the six treatment groups for each species using ANOVA (blocked by location in the greenhouse) followed by post-hoc Tukey HSD tests. All analyses were conducted in SPSS 24.0 with α = 0.05.

### Seed germination experiment

Seed germination of corn (field corn, Carolina Biological Supply) and tomato (cv. Beefsteak, Plantation Products) was evaluated over an 18-day period in March 2017. Four randomly selected seeds of each species were placed between paper towels in 60 mm petri dishes, with 3 petri dishes per treatment group for each species. This resulted in a total of 18 petri dishes for each species, with 12 seeds per treatment group.

Initially, to saturate the paper towels, all petri dishes received 5 ml of the appropriate erythritol concentration on 12 March 2017 at 09:00 hrs. To keep the paper towels sufficiently moist, an additional 1 ml of the appropriate solution was added to each plate every subsequent day at 09:00 hrs. The seeds were monitored twice per day (at 09:00 hrs and 15:00 hrs) until germination was observed. Germination was defined as the emergence of the radicle from the seed coat. The covered petri dishes were stored on a laboratory bench at room temperature throughout the experiment.

Percent germination and mean time to germination (in days) were calculated for each petri dish (n = 3 replicate petri dishes per treatment group for each species; S1 Dataset). The percent germination datasets for both corn and tomato grossly violated normality and heteroscedasticity assumptions, so a Kruskal-Wallis test (nonparametric alternative to an ANOVA) was used, followed by post-hoc pairwise Mann-Whitney U tests, to examine differences in median percent germination among the treatment groups. Mean days to germination was compared among the treatment groups for both corn and tomato using ANOVA followed by post-hoc Tukey HSD tests. All analyses were conducted in SPSS 24.0 with α = 0.05.

## Results

### Erythritol reduces tomato dry weight, but has no effect on corn dry weight

**Fig 1:**
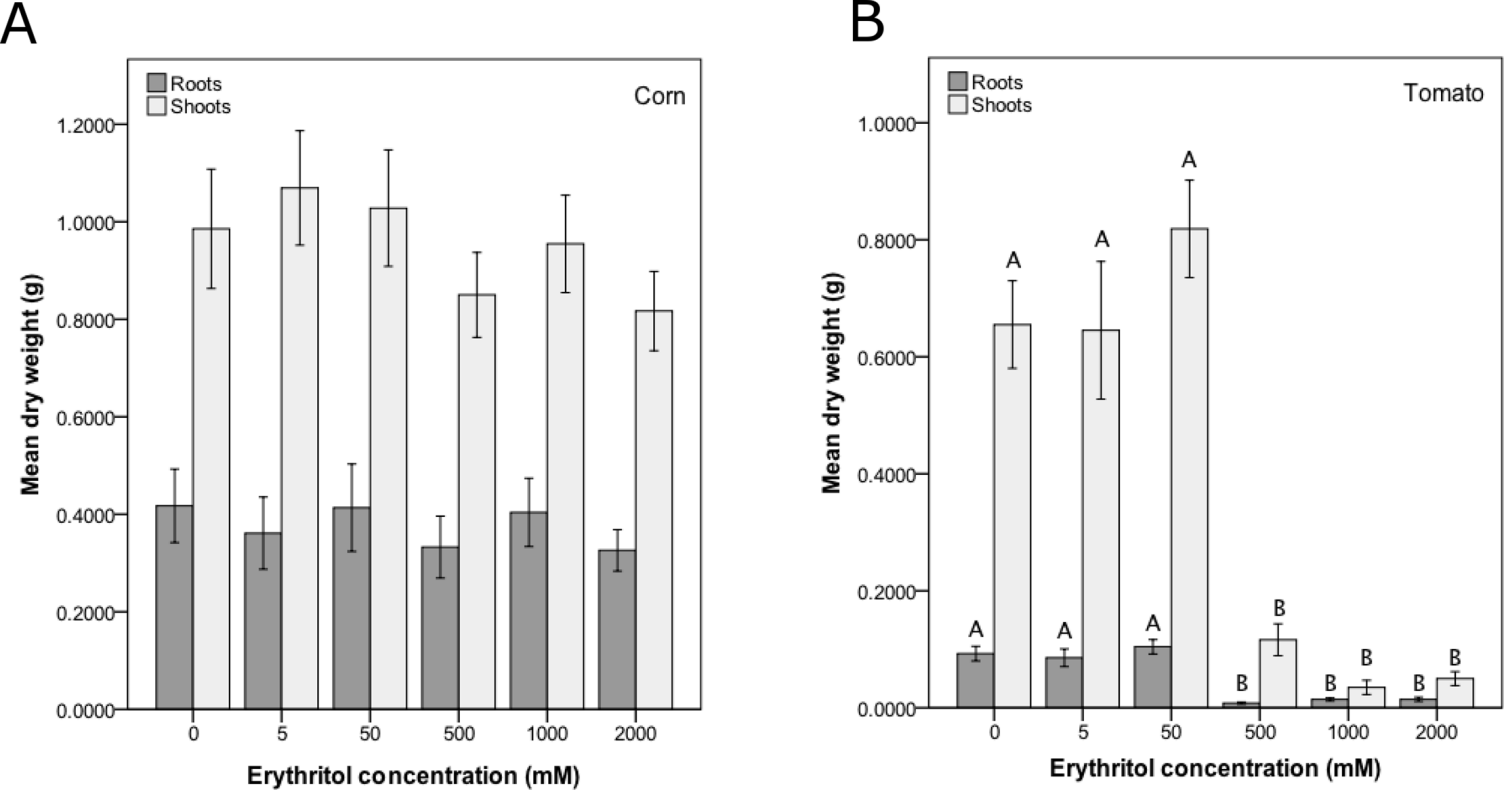
Erythritol reduces tomato dry weight, but has no effect on corn dry weight. Mean (± 1 SE) root and shoot dry weight (biomass) for A) corn and B) tomato seedlings treated with erythritol spray at 6 different concentrations for 21 days (corn) or 28 days (tomato). There were 11 plants of each species per erythritol treatment group. Means with the same letter within a response variable are not significantly different from each other (Tukey HSD test, p < 0.05).

Corn root and shoot dry weight (g) were not significantly different among the erythritol treatment groups (ANOVA, Root: F_5,50_ = 0.7, p = 0.645; Shoot: F_5,50_ = 1.3, p = 0.262; Table 2, Fig 1). In contrast to these results for corn, both tomato root and shoot dry weight were significantly different among the erythritol treatment groups (ANOVA, Root: F_5,50_ = 26.7, p < 0.001; Shoot: F_5,50_ = 31.8, p < 0.001; Fig 1). Mean tomato root and shoot dry weight were not significantly different among the control group and the two lowest erythritol treatment groups (5 and 50 mM; Table 2, Fig 1). Tomato root and shoot dry weight were significantly lower in the 500, 1000, and 2000 mM groups than in the control group and the two lowest erythritol concentration groups (5 and 50 mM). The tomato root and shoot dry weight were not significantly different among these three highest erythritol treatment groups (500, 1000, and 2000 mM).

**Table 2:** Seedling growth (measured as dry weight) and seed germination of corn and tomato exposed to 5 different erythritol treatments and a deionized water control. Dry weight: n = 11 replicates per treatment group for each species; seed germination: n = 3 replicate petri dishes (with 4 seeds per petri dish) per treatment group for each species.

Erythritol concentration (mM)	Mean (± 1 SD) root dry weight (g)	Mean (± 1 SD) shoot dry weight (g)	Median germination (%)	Mean (± 1 SD) days to germination
**Corn**				
0	0.417 ± 0.25	0.986 ± 0.41	100	2.6 ± 0.3
5	0.361 ± 0.25	1.069 ± 0.39	100	2.8 ± 0.3
50	0.413 ± 0.30	1.028 ± 0.40	100	3.4 ± 0.1
500	0.333 ± 0.21	0.850 ± 0.29	100	3.9 ± 0.3
1000	0.404 ± 0.23	0.955 ± 0.33	50	6.3 ± 1.5
2000	0.326 ± 0.14	0.817 ± 0.27	0	—
**Tomato**				
0	0.093 ± 0.04	0.655 ± 0.25	100	3.2 ± 0.1
5	0.085 ± 0.05	0.645 ± 0.39	100	3.3 ± 0.4
50	0.104 ± 0.04	0.819 ± 0.28	50	3.5 ± 0.5
500	0.008 ± 0.01	0.116 ± 0.09	0	—
1000	0.014 ± 0.01	0.035 ± 0.04	0	—
2000	0.014 ± 0.01	0.050 ± 0.04	0	—

### Erythritol causes dose-dependent inhibition of germination in corn and tomato

**Fig 2:**
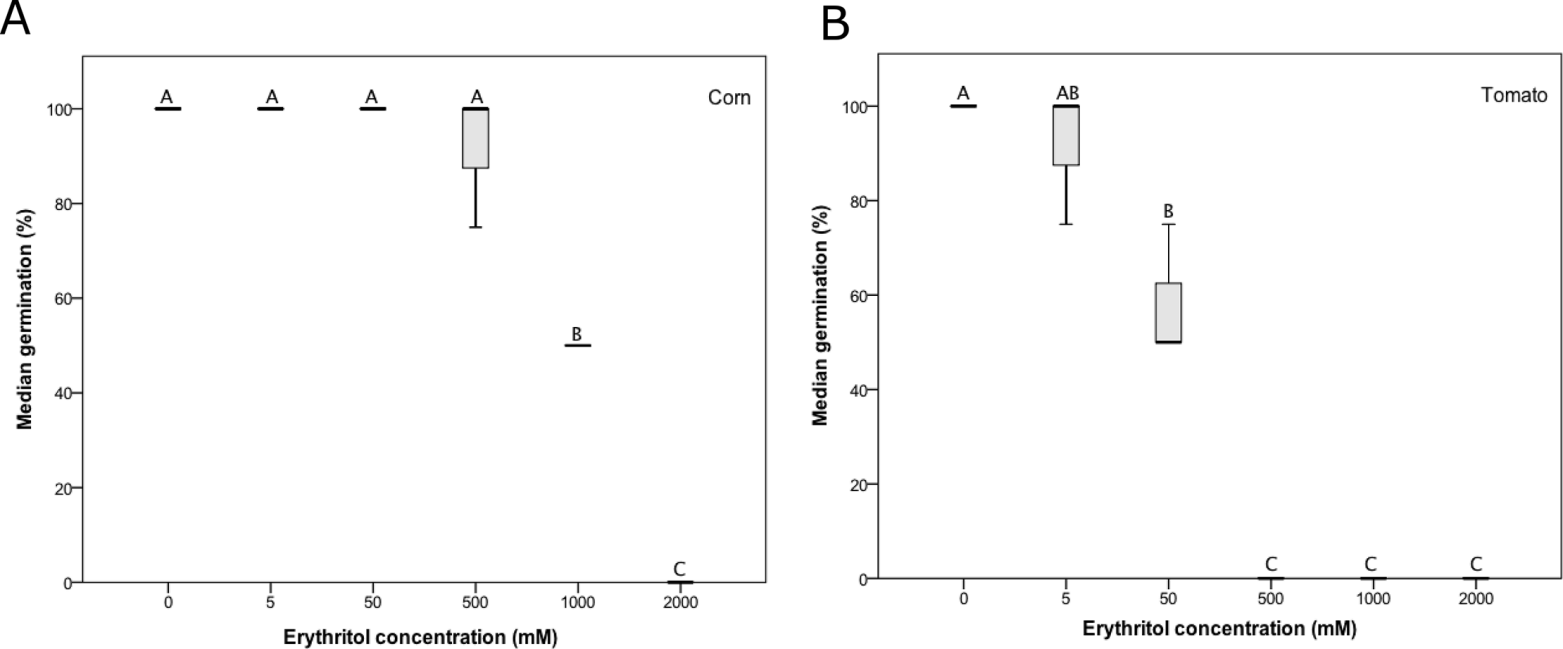
Erythritol causes dose-dependent inhibition of seed germination in corn and tomato. Median (IQR) A) corn and B) tomato seed percent germination over 18 days at 6 different treatment concentrations of erythritol. For each species, seeds were treated in petri dishes (4 seeds per dish; 3 dishes per treatment group) and the percent germination was calculated for each petri dish. Medians with the same letter are not significantly different from each other (Mann-Whitney U test, p < 0.05).

Erythritol negatively affected corn seed germination. Median corn seed germination (%) was significantly different among the treatment groups (Kruskal-Wallis, df = 5, χ^2^ = 15.9, p = 0.007). There was no difference in median germination among the control, 5 mM, 50 mM, and 500 mM erythritol treatment groups, but germination was significantly lower in the 1000 mM group than in the previous four groups. No seeds germinated in the 2000 mM group, and this group’s median germination was significantly lower than all other groups (Table 2, Fig 2).

**Fig 3:**
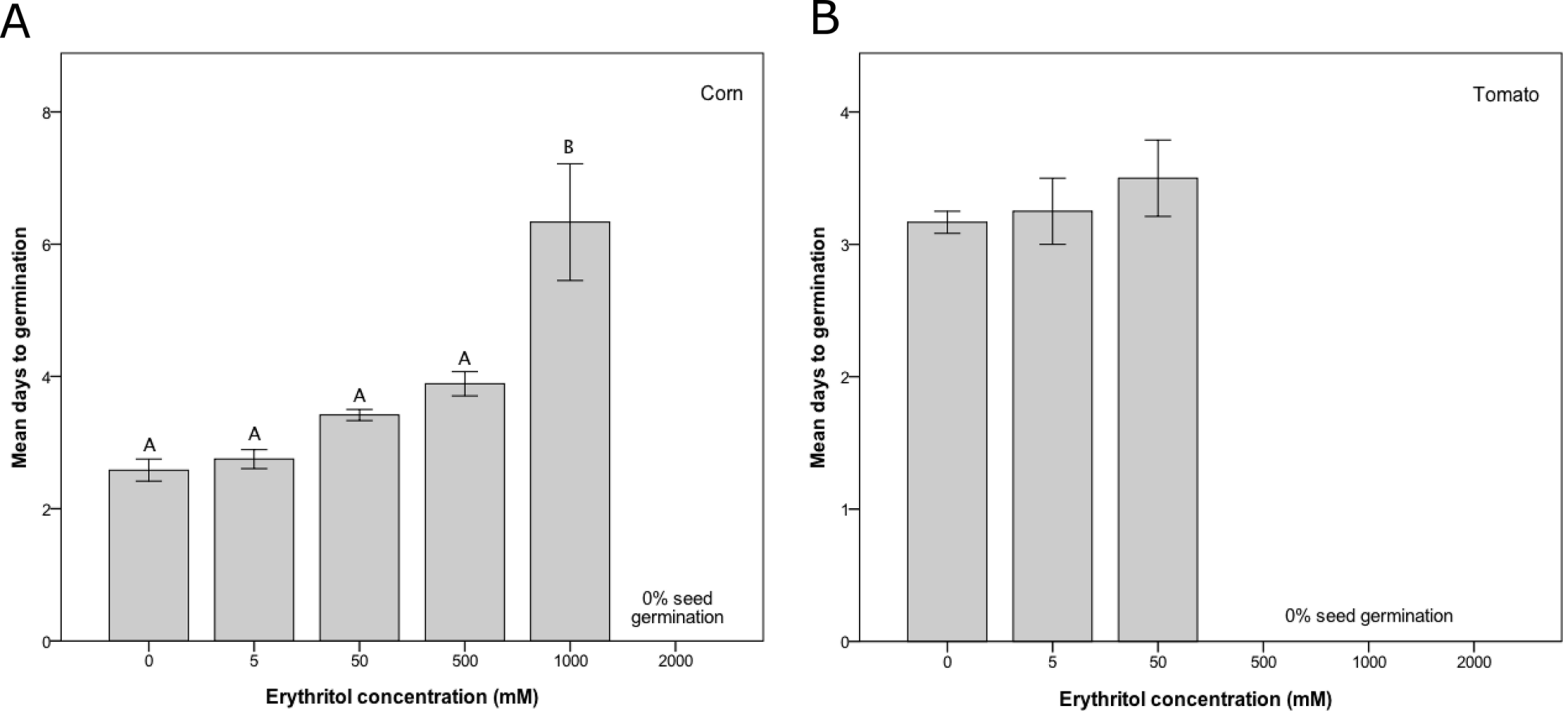
Erythritol delays seed germination in corn. Mean (± 1 SE) number of days to seed germination for A) corn and B) tomato over 18 days at 6 different treatment concentrations of erythritol. For each species, seeds were treated in petri dishes (4 seeds per dish; 3 dishes per treatment group) and the mean days to germination was calculated for each petri dish. Note that no seeds germinated in the 2000 mM treatment group for corn and the 500 mM, 1000 mM, and 2000 mM treatment groups for tomato, so these treatment groups were not included in the analysis. Means with the same letter are not significantly different from each other (Tukey HSD test, p < 0.05).

The mean time to corn seed germination was significantly different among the control, 5 mM, 50 mM, 500 mM, and 1000 mM treatment groups (the 2000 mM treatment group was excluded from this analysis because no seeds germinated; ANOVA F_4,10_ = 13.2, p = 0.001). Mean time to germination was significantly delayed in the 1000 mM group compared to the control, 5 mM, 50 mM and 500 mM groups, and the latter four groups were not significantly different from each other (Table 2, Fig 3).

Similarly, erythritol also inhibited the germination of tomato seeds, which appeared to be more sensitive than the corn seeds. Median tomato seed germination (%) was significantly different among the treatment groups (Kruskal-Wallis, df = 5, χ^2^ = 16.6, p = 0.005; Fig 2). There was no difference in median germination between the control and 5 mM erythritol treatment groups, but germination was significantly higher in the control group than the 50 mM erythritol treatment group and those groups in which germination was completely inhibited (500, 1000, and 2000 mM groups; Table 2, Fig 2). Median germination was not significantly different between the 5 mM and 50 mM groups, but both of these groups had significantly higher median germination than the 500, 1000, and 2000 mM groups.

Among the three treatment groups in which tomato seeds germinated (control, 5 mM, and 50 mM groups), there was no significant difference in time to germination (ANOVA, F_2,6_ = 0.6, p = 0.583; Table 2, Fig 3). However, there was a trend towards longer germination times with higher erythritol concentrations (Fig 3).

## Discussion

Our results suggest that some agricultural crops may be damaged by erythritol spray that is concentrated enough to kill insect pests (Table 1). Furthermore, our results highlight other potential applications of erythritol as an organic herbicide.

### Erythritol reduces tomato plant dry weight, but has no effect on corn plants

Erythritol caused dose-dependent inhibition of belowground and aboveground growth in tomato seedlings (Table 2, Fig 1), with significantly reduced root and shoot dry weight at and above the typical minimum insecticidal dose of 500 mM (Table 1). This concentration of erythritol is at or below the typical insecticidal dose for eggs, larvae (500 mM), and adults (1750–2000 mM) of *D*. *suzukii* in the field [11,15] (Table 1), as well as adult *M*. *domestica* (500 mM) [18] and *D*. *melanogaster* (500 mM) [8,11] (Table 1). However, although erythritol inhibited the growth of tomatoes, it did not affect corn growth, even at the highest treatment concentration (2000 mM; Fig 1).

We hypothesize that the “Lotus-Effect” [22] is important to understanding this striking difference in response to erythritol by corn and tomato plants. The lotus effect has been observed on water-repellant plant leaves, which are self-cleaning in the sense that any particles on these leaves are quickly removed when the leaves experience precipitation [22–24]. We observed that the erythritol spray remained on the tomato plant leaves after treatment, whereas the spray appeared to drip off the corn leaves. Corn plant leaves grow more vertically than tomato plant leaves, and this architectural difference may have facilitated the erythritol spray dripping off the corn plants. Additionally, physical and/or chemical differences between the two species in their leaf epidermal characteristics, including the cuticle and the trichomes (leaf hairs), may have been responsible for greater accumulation and penetration of the water-based erythritol solutions into the tomato plants than the corn plants. This reasoning is supported by our finding that corn seeds, which were constantly exposed to erythritol solution, were affected by erythritol (Figs 2 and 3) whereas the corn plants were not (Fig 1). Although previous research has shown that corn plants treated with the 6-carbon polyol mannitol showed improved growth and physiology under conditions of salt-stress, the mannitol was applied with a surfactant (Tween 20) [25] that may have allowed it to adhere to and penetrate the corn cuticle more effectively. In contrast, our erythritol spray contained no adjuvants.

Possibly exacerbating the morphological differences between the two crop species, the tomato plants grew more slowly than the corn plants (see the control group means in Fig 1), so the tomato plants experienced increasingly greater erythritol dosage per unit leaf area over time. Nevertheless, the negative effects on the tomato seedling growth were not limited to the last couple of weeks of growth, but were apparent very early on. The tomato plants also experienced 7 more days of growth (28 days versus 21 days for the corn; see Methods), and it is possible that this longer duration of exposure may have further harmed the plants. However, given that there is no sign of a dose-dependent response trend in the corn growth (Fig 1), it seems unlikely that this longer growth period explains the marked differences in response observed between corn and tomato.

Given that erythritol is water soluble, it is unlikely to accumulate in the soil in drained pots in the greenhouse or in well-watered agricultural fields. In our study, an 18-hour period occurred between our erythritol application (15:00 hrs) and plant irrigation (09:00 hrs). Even though this 18 hour time period mostly occurred overnight, it seems sufficient to elicit a plant root response to erythritol in the soil. However, while some polyols seem to be most active in sink tissues like roots [5,26], a previous study on the model plant *Arabidopsis* found that roots are less sensitive to certain polyols than are shoots [27]. Other plant roots, like corn, may likewise be mostly unaffected by erythritol in the soil. In the case of tomato, it is possible that the reduced growth belowground (Fig 1) was due to erythritol exposure in the soil, but it is equally likely that aboveground reductions in photosynthesis resulted in less carbon availability for root growth.

The accumulated erythritol residue on the tomato leaves may have had negative effects on photosynthesis or other physiological processes either through direct physical processes, i.e., smothering the leaves [28], and/or by altering or suppressing certain homeostatic and biochemical pathways. Polyols can be produced as compatible solutes to respond to stressful conditions (e.g., salt and drought stress) [26], but they can be detrimental to normal plant cellular activity if they accumulate to high levels, ultimately resulting in reduced growth (e.g., [5]). In insects, erythritol is believed to pass, unaltered, through the midgut and into the hemolymph, causing a sustained increase in osmotic pressure that ultimately leads to insect death, perhaps through starvation or cellular desiccation [11,13,16]. Similar changes to osmotic gradients might be expected to occur in plant leaf cells, resulting in desiccation. Indeed, we noted obvious desiccation of the leaves of erythritol-treated tomato plants. In addition or alternatively, exogenous erythritol may act as a substrate analog inhibitor of an enzyme in the 2-C-methyl-D-erythritol 4-phosphate (MEP) pathway that produces isoprenoids in plastids [29]. Similarly, some herbicides target enzymes in the MEP pathway [30]. These erythritol-induced direct and/or indirect effects on plant physiology likely resulted in reduced nutrition and ultimately reduced tomato growth both belowground and aboveground.

### Erythritol inhibits corn and tomato seed germination

Erythritol caused dose-dependent inhibition of seed germination in both corn and tomato (Table 2, Fig 2) and delayed germination in corn (Table 2, Fig 3). Germination was reduced in both crops at 1000 mM erythritol, which is below the typical insecticidal dose (1750–2000 mM) for adult *D*. *suzukii* in the field [11,15] (Table 1). Furthermore, at the typical minimum insecticidal dose (500 mM; Table 1), tomato seed germination was completely inhibited relative to the water control (Table 2; Figs 2 and 3). Our results suggest that tomato seeds are more sensitive than corn seeds to application of exogenous erythritol, a pattern that we also observed in the tomato and corn seedlings (Fig 1). This pattern may indicate that species-specific characteristics, similar to those discussed above for seedlings, may underlie erythritol sensitivity differences among species.

The total erythritol dose over time was likely greater for seeds than plants, because the seeds were constantly exposed to the erythritol solution, which was prevented from draining out of the enclosed environment. This greater intensity of erythritol accumulation and exposure may help to explain why the corn seeds responded to high concentrations of erythritol (≥1000 mM) but the corn plants did not (Figs 1–3).

It is worth noting that a post-experimental observation of our corn and tomato seeds from the 2000 mM treatment group indicated that many of the seeds were still viable despite not germinating. These seeds were placed on new paper towels moistened with water on 30 March, and the majority had germinated by 3 April (8 of 11 tomato seeds and 6 of 12 corn seeds had germinated). This small trial suggests that erythritol may be inhibiting seed germination by altering or suppressing biochemical pathways that are necessary for seeds to germinate. In a previous study, *Arabidopsis* seeds that were exogenously treated with two 6-carbon polyols, sorbitol and mannitol, at 150 mM also exhibited delayed germination [27]. At the same time, erythritol’s reported antimicrobial properties [11,31] may have reduced rotting of the seeds.

### Erythritol use in agricultural settings

Extrapolating our results to an agricultural setting is difficult given the limited studies on erythritol’s effects on plants and other non-target organisms. At this early stage, most of the published studies on erythritol’s insecticidal characteristics have been conducted in a lab setting, without treating crop plants and without looking for non-target effects on other organisms. Only two published studies have applied erythritol-based spray directly to crop plants [15,16]. One showed that a thrice-applied 500 mM erythritol, Truvia-based spray killed *D*. *suzukii* larvae on rabbiteye blueberry (*Vaccinium virgatum*) and blackberry (*Rubus* sp.) bushes in a field setting [15]. The other study found that a single erythritol spray of 500 mM or 2000 mM, followed by 6 days of water spray, killed *D*. *suzukii* adults on southern highbush blueberry plants in a greenhouse [16]. Neither study mentioned any negative non-target effects of the erythritol sprays on the blueberry or blackberry plants. In fact, Sampson et al. [15] suggested that erythritol’s antimicrobial activity (e.g., [31]) may protect sprayed plants from disease.

Contrary to this suggestion, we did not see any evidence of beneficial effects of erythritol spray to crop plants or seeds in our study, even at lower doses that likely would be too dilute for insecticidal use. That said, our daily treatment of both plants and seeds was almost certainly more extreme than is likely to occur in a field setting, at least for insecticidal use. In the case of *D*. *suzukii*, for example, erythritol is likely to be sprayed on plants every several days (e.g., 3 to 5 times over several weeks) rather than daily [15]. On the other hand, rainy weather may require that insecticidal erythritol spray be applied more often. Furthermore, if the goal is to kill adult *D*. *suzukii*, a 2000 mM spray may be needed in the field [11], with more potential for negative effects to crop plants and seeds at this higher concentration. Multiple studies have called for the use of erythritol under low humidity to improve its effectiveness at killing insect pests [11,15]. Under low humidity, the water in the erythritol spray should evaporate more quickly, leaving behind a potentially detrimental residue on the leaves [28] that will not be washed off even if the plants are not treated daily. Different temperature and light regimes in a field setting may also modulate erythritol’s biological effects on both target pests as well as non-target crops.

The only erythritol spray that has been used in the field so far was a mix of Truvia Baking Blend, water, and Dawn hand-soap [15]. As further field trials are designed, various adjuvants like Dawn soap are likely to be added to the insecticidal erythritol spray. Our results for corn and tomato plants suggest that certain leaf epidermal characteristics may be protective against erythritol, so insecticidal adjuvants that allow for greater erythritol penetration into the internal leaf tissues may result in damage to naturally resistant crop plants (e.g., corn, mature berry bushes) particularly with long-term use. It is also possible that erythritol’s effects on crops may be altered by the additional ingredients in Truvia, such as stevia leaf extract [6].

### Erythritol use as an herbicide

The observed extremely negative effects of higher concentrations of erythritol on our tomato seedlings and both tomato and corn seeds suggest that erythritol may be effective as an organic herbicide, possibly by inhibiting the MEP pathway discussed above. As a pre-emergent herbicide, erythritol is unlikely to completely kill weed seeds because we observed that at least some of the tomato and corn seeds remained viable despite delayed germination (see above). However, erythritol could be used to delay weed seed germination to allow transplanted crop seedlings a head start on growth. Since it is water soluble, it would be particularly useful during times of reduced precipitation.

Erythritol also has potential as a post-emergent herbicide. It is interesting that the broad-leaved dicotyledonous tomato plants were greatly negatively affected by moderate concentrations of erythritol, whereas the monocotyledonous corn plants were not affected even by high concentrations of erythritol. This finding suggests that erythritol may have use as a post-emergent herbicide against broad-leaved weeds in lawns or in fields containing grain crops. Alternatively, erythritol may have use as a generalized herbicide if combined with an adjuvant that allows it to penetrate through the leaf epidermis of a wide variety of weed plants. Because the cuticle changes as plants mature [24,32,33], we propose that seedlings might be more susceptible to erythritol than adult plants.

Future research in this area should first work to evaluate which treatment timing intervals and concentrations of erythritol, both with and without adjuvants, are damaging to common weeds at multiple life stages. Next, experimental field studies can be used to evaluate whether erythritol kills weeds without damaging crops or other desirable plant species, such as lawn grasses.

### Conclusions

Given the results of this study, we recommend that future field studies monitor any effects of insecticidal erythritol sprays on the non-target crop plants as well as the target pests. Our results suggest that we can expect variable responses of plant crops to erythritol, depending not only on the concentration of erythritol but also on the plant life stage and a particular species’ morphological, physiological, and biochemical characteristics. Importantly, susceptible plant crops may be harmed at the moderate to high concentrations of erythritol that appear to be needed for effective insecticidal formulations. If the crops are found to be negatively affected by erythritol spray, then bait traps, which have been suggested by previous studies [8,11], may present a viable alternative. We also propose that erythritol may have promise as an organic herbicide.

## Supporting information

S1 Dataset Supporting dataset for this study. Supporting data are provided in an .xlsx file. The first spreadsheet in the file contains root and shoot dry weight (g) data for both corn and tomato; the second spreadsheet contains seed germination data for both corn and tomato. Germination data are presented as the percent and mean days to germination for the 4 seeds per petri dish (3 petri dishes per erythritol treatment group per crop species).
(XLSX)
